# Microcirculatory disturbances in type 2 diabetes: early detection using laser doppler flowmetry for personalized care

**DOI:** 10.3389/fendo.2025.1651525

**Published:** 2025-09-25

**Authors:** Zlatoslava O. Shaienko

**Affiliations:** Department of Endocrinology and Pediatric Infectious Diseases, Poltava State Medical University, Poltava, Ukraine

**Keywords:** diabetes mellitus, microcirculation, laser doppler flowmetry, microvascular disorders, endothelial dysfunction

## Abstract

**Introduction:**

Type 2 diabetes mellitus (T2DM) is frequently associated with microvascular dysfunction that contributes to the development of complications such as neuropathy, nephropathy, and retinopathy. Early detection of these alterations is essential for effective prevention and personalized care.

**Aim:**

To evaluate microcirculatory changes in patients with type 2 diabetes using laser Doppler flowmetry (LDF).

**Materials and methods:**

This observational study included 25 individuals: 19 patients with T2DM and 6 healthy controls. Microcirculatory parameters were assessed using LDF, a non-invasive technique for evaluating perfusion in real time. Parameters such as endothelial nitric oxide–dependent activity, neurogenic and myogenic regulation, oxidative metabolism, and nutritive blood flow were measured and analyzed.

**Results:**

Patients with T2DM demonstrated significant microcirculatory disturbances, including reduced endothelial NO-dependent activity, increased sympathetic adrenergic and myogenic activity, and elevated oxidative stress levels. A decreased coefficient of variation and increased nutritive flow suggested a compensatory adaptation of vascular tone regulation. A strong positive correlation was identified between the microcirculation index and regulatory system tension (r = 0.7561; p = 0.0002), indicating systemic vascular strain.

**Conclusion:**

LDF proved to be an effective tool for the early detection of subclinical microvascular changes in patients with T2DM. These findings support the incorporation of LDF into clinical protocols for individualized risk stratification, early intervention, and treatment monitoring. Further longitudinal studies with larger cohorts are warranted to validate its role in precision diabetology.

## Introduction

Diabetes mellitus is one of the most prevalent chronic endocrine disorders, significantly affecting patients’ quality of life. This disease is characterized by the development of microvascular complications such as diabetic retinopathy, nephropathy and neuropathy (1–4). Microcirculatory impairment plays a key role in the pathogenesis of these complications, as it is at this level that disturbances in blood flow and tissue gas exchange occur (1, 4, 5).

The study of the microcirculatory system is of great importance for the timely diagnosis and monitoring of the progression of diabetes-related complications (6–8). One of the up-to-date non-invasive methods for assessing microcirculation is LDF (9–11). This technique can be applied to the surface of any organ, most commonly on the skin or mucous membranes. In recent years, LDF has been the subject of numerous publications due to its ease of use, short measurement duration and the minimal training required for the operator. This method enables the evaluation of the functional state of the microvascular system by analyzing blood flow in real time, making it highly valuable in clinical practice (12, 13). LDF is a state-of-the-art, highly sensitive method for investigating the microcirculatory system and can be effectively utilized in diagnosing circulatory disorders in patients with diabetes mellitus. The method is based on analyzing changes in the frequency of laser radiation reflected from erythrocytes moving through microvessels. LDF can detect subclinical disturbances even in the early stages, when clinical symptoms may still be absent (9–13).

## Materials and methods

This observational study included 25 patients aged 40–66 years (mean age: 58 ± 1.7 years) treated at the Endocrinology Department of the 2nd City Clinical Hospital in Poltava between March and August 2023. The study population was divided into two groups: the control group (n = 6), which included individuals without diabetes mellitus, and the study group (n = 19), composed of patients diagnosed with T2DM.

All participants provided written informed consent prior to inclusion in the study. The study protocol was approved by the Biomedical Ethics Committee of Poltava State Medical University (Protocol No. 235, dated February 20, 2025). This research is part of the broader project “Development of Means for the Correction of Pathological Changes in the Digestive System in the Context of Civilization-Related Diseases” (State registration number: 0124U001922; 2024–2028).

Microcirculatory assessment of the lower limbs (dorsal foot) was performed using the LASMA PF laser Doppler flowmeter. Measurements were taken in a resting state over a 6-minute period. The data were recorded as perfusion curves. The microcirculation index (MI) was calculated to reflect baseline tissue perfusion.

In addition to absolute perfusion, we evaluated the regulatory mechanisms influencing blood flow by analyzing both active and passive rhythmic oscillations in the LDF signal. Active components included endothelial (Ae), neurogenic (An), and myogenic (Am) activity, while passive components were associated with respiratory (Ad) and cardiac (Ac) influences. Functional adaptation of the vascular system was assessed using the coefficient of variation (Kv). The oxidative metabolism index (OMI) and nutritive blood flow (Mntr) were also calculated.

Wavelet analysis was used to decompose the LDF signal and quantify the contributions of the various frequency ranges corresponding to the described mechanisms. This enabled a comprehensive evaluation of the functional integrity of the microvascular regulatory system.

Statistical analysis was conducted using GraphPad Prism 5.03 and Microsoft Excel 2010. Normality of distribution was tested using the D’Agostino–Pearson omnibus test and Kolmogorov–Smirnov test. For normally distributed data, results are reported as mean ± standard error of the mean (M ± m); non-normal data are presented as median [25th–75th percentiles]. Between-group comparisons were performed using the Kruskal–Wallis test. For paired comparisons, the Student’s t-test or Wilcoxon signed-rank test was applied, depending on data distribution. Correlations between continuous variables were analyzed using Pearson’s or Spearman’s correlation coefficients. A p-value < 0.05 was considered statistically significant.

## Results

The mean age of patients in the study group was 61.47 ± 2.196 years, the average duration of diabetes mellitus was 11.79 ± 1.871 years, and the mean age at onset of diabetes was 49.68 ± 2.378 years (**Table 1**). No statistically significant difference was found between the study and control groups in terms of sex distribution (p=0.0756).

**Table 1:** Characteristics of patients in the study and control groups.

Parameter	Study group (before treatment)	Control group	р
n	19	6	–
Men/women, (n/n)	12/7	3/3	р = 0,0756
Age, years	61.47± 2.196	51 ± 4.726	р < 0.0001
Age at diabetes onset (years)	49.68 ± 2.378	–	–
Duration of diabetes (years)	11.79 ± 1.871	–	–
HbA1c (%)	8.967 ± 0.3962	–	–
BMI (kg/m²)	30.21 ± 1.129	–	–

The analysis of comorbidities and complications in patients from the study group is presented in **Table 2**. A family history of diabetes mellitus was noted in 31.6% of patients, indicating a genetic predisposition to the development of this disease.

**Table 2:** Comorbidities in patients of the study group.

Comorbidities	Study group (n/%)
Family history	6 (31.6%)
Myocardial infarction	2 (10.5%)
Stroke	1 (5.3%)
DM complications	
- nephropathy	4 (21.1%)
- angiopathy	13 (68.4%)
- neuropathy	19 (100%)
- retinopathy	2 (10.5%)
MAU, mg/day	10.5 [4.2; 15.20]

Among cardiovascular events, 10.5% of patients experienced a myocardial infarction, while stroke was documented in 5.3% of cases.

Complications of diabetes mellitus were highly prevalent in the study group. The most common complication was neuropathy, observed in all patients (100%). Angiopathy was identified in 68.4% of patients, while nephropathy was present in 21.1%. Diabetic retinopathy was documented in 10.5% of patients.

Additionally, the level of microalbuminuria (MAU) was assessed, which serves not only as a marker of renal impairment but also indicates an increased risk of cardiovascular complications. The mean MAU level in patients was 10.5 mg/day, with an interquartile range of [4.2; 15.20], suggesting the presence of early signs of renal dysfunction in a subset of patients. These findings underscore the high prevalence of micro- and macrovascular complications in patients with diabetes mellitus, highlighting the need for active monitoring and timely intervention to prevent their progression.

**Table 3** presents the results of LDF, characterizing microcirculatory activity and the functioning of regulatory mechanisms in patients with type 2 diabetes mellitus (study group) compared to healthy individuals (control group). According to the obtained data, all LDF parameters differed significantly between the study and control groups (p<0.05).

**Table 3:** LDF Parameters in patients of the study and control groups.

LDF Parameters	Study group	Control group	р
Endothelial NO-dependent activity, Ae	1.263 ± 0.2034	1.308 ± 0.2657	р = 0.0044
Neurogenic sympathetic adrenergic activity, An	1.286 ± 0.1849	1.0 ± 0.1602	р = 0.0015
Myogenic activity, Am	1.638 ± 0.2193	1.372 ± 0.2703	р = 0.0039
Passive frequency range of respiratory rhythm, Ad	2.042 ± 0.2977	1.808 ± 0.3833	р = 0.0053
Passive frequency range of cardiac rhythm,	2.463 ± 0.3684	2.402 ± 0.5453	р = 0.0070
Functional tension of regulatory systems assessed by the coefficient of variation, Kv	10.28 ± 0.7712	15.30 ± 1.429	р < 0.0001
Oxidative metabolism index, OMI	8.670 [3.32; 12.04]	4.073 ± 1.301	р = 0.0313
Mean value of the microcirculation index, M	52.53 ± 6.278	34.28 ± 3.645	р = 0.0002
Mean nutritive blood flow, Mntr	23.16 ± 2.607	14.04 ± 1.804	р = 0.0006

*the difference was statistically significant when compared to the control group (p < 0.05).

The status of blood flow in the microvessels of the foot skin was evaluated by assessing the mean perfusion (M), which was 34.27 ± 3.64 perfusion units (PU) in the control group and 52.53 ± 6.27 PU in the study group. Thus, higher microcirculation index values were observed in patients with diabetes mellitus compared to controls. This finding can be explained by the impact of prolonged neuropathy on microcirculatory function, leading to increased cutaneous blood flow.

The endothelial NO-dependent activity index (Ae) in the study group was 1.263 ± 0.2034, which was significantly lower than that in the control group (1.308 ± 0.2657, p = 0.0044). This indicates a reduced capacity of the endothelium to synthesize nitric oxide (NO), thereby impairing vascular regulatory function.

Neurogenic sympathetic adrenergic activity (An) in patients with T2DM was elevated (1.286 ± 0.1849) compared to the control group (1.0 ± 0.1602, p=0.0015). This indicates hyperactivity of the sympathetic nervous system, which affects microcirculatory tone.

In the study group, the value of myogenic activity (Am) was significantly higher (1.638 ± 0.2193) compared to the control group (1.372 ± 0.2703, p=0.0039), indicating enhancement of myogenic regulation as a compensatory mechanism.

The study group exhibited significantly lower values of the coefficient of variation (Kv) (10.28 ± 0.7712) compared to the control group (15.30 ± 1.429, p<0.0001), indicating a high level of regulatory system stress in diabetes mellitus.

The oxidative metabolism index (OMI) showed a significantly higher median level in the study group (8.670 [3.32; 12.04]) compared to the control group (4.073 ± 1.301, p = 0.0313), indicating increased oxidative stress in patients with diabetes mellitus.

The nutritive blood flow index (Mntr) was significantly higher in the study group (23.16 ± 2.607) compared to the control group (14.04 ± 1.804, p=0.0006), reflecting compensatory activity aimed at providing tissues with energy.

Thus, patients with T2DM exhibit significant alterations in microcirculatory function, including reduced endothelial activity, increased sympathetic and myogenic activity, as well as elevated oxidative stress. These changes result from impaired regulation of vascular tone and metabolic imbalance characteristic of this disease. This underscores the importance of early monitoring of microcirculatory status in such patients.

The analysis of correlation relationships between microcirculation parameters and clinical characteristics of patients with diabetes mellitus in the study group revealed the following results in **Table 4**.

**Table 4:** Correlation relationships in patients with diabetes mellitus of the study group.

Parameters					
	Mean value of the microcirculation index, M	Functional tension of regulatory systems assessed by the coefficient of variation, Kv	Endothelial NO-dependent activity, Ae	Myogenic activity, Am	Mean nutritive blood flow, Mntr
Diabetes mellitus onset, age (years)	r= -0.02598; p= 0.9159				
Duration of diabetes, years	r=-0.2243; p= 0.3558	r= -0.1965; p= 0.4201	r=-0.2412; p= 0.3199	r=-0.3112; p= 0.1946	r=-0.1756; p= 0.4721
Mean value of the microcirculation index, M			r=0.7561; p= 0.0002		

The analysis of the correlation relationships between microcirculation parameters and clinical characteristics in patients with T2DM revealed several notable findings. No significant correlations were found between the age of diabetes onset and parameters of microcirculation, regulatory systems, or endothelial activity (r = –0.02598; p = 0.9159), indicating that the age at which the disease begins does not have a meaningful impact on these physiological markers. Furthermore, the duration of diabetes was not significantly associated with key microvascular indicators, including the mean microcirculation index (r = –0.2243; p = 0.3558), functional tension of regulatory systems (Kv) (r = –0.1965; p = 0.4201), endothelial NO-dependent activity (Ae) (r = –0.2412; p = 0.3199), myogenic activity (Am) (r = –0.3112; p = 0.1946), or nutritive blood flow (Mntr) (r = –0.1756; p = 0.4721). These weak and statistically non-significant relationships suggest that disease duration is not a primary determinant of microcirculatory changes in this cohort. In contrast, a strong positive correlation was observed between the mean microcirculation index (M) and the functional tension of regulatory systems (Kv), with r = 0.7561 and p = 0.0002. This indicates that increased microcirculatory activity is associated with a significant rise in regulatory load, likely reflecting compensatory adaptation mechanisms within the microvascular network in response to chronic vascular stress.

## Discussion

Laser Doppler flowmetry is a state-of-the-art non-invasive method for studying microcirculation that finds applications across various medical specialties (11–13). This technique is utilized to evaluate skin microcirculation and has demonstrated clinical efficacy in rheumatology, dermatology, surgery and diabetology. In surgical practice, LDF aids in evaluating the severity of burns and facilitates decision-making regarding the initiation of therapy. The method is also effective for monitoring wound healing, including postoperative wounds. In rheumatology, LDF is employed for diagnosing conditions such as Raynaud’s phenomenon and systemic scleroderma. In dermatology, it is used to assess the severity of psoriasis and to evaluate treatment efficacy.

LDF holds particular value in diagnosing microcirculatory disturbances in patients with type 1 and type 2 diabetes mellitus (14, 15). This method is indispensable for this patient group because pathological changes in microcirculation occur earlier than the development of severe microangiopathic lesions. Timely detection of such changes allows for the implementation of preventive measures or the adaptation of therapy aimed at reducing the impact of hyperglycemia on the vasculature (16). Early intervention helps reduce the risk of progression of microvascular complications, prevent systemic consequences of diabetes, and significantly improve patients’ quality of life. Early diagnostic methods, such as LDF, enable the assessment of vascular responses to therapy, timely modification of treatment strategies, and ongoing monitoring of treatment efficacy (16). Considering its versatility, non-invasiveness and high sensitivity, LDF holds promise to become a standard tool for early diagnosis and monitoring of microcirculatory status across various clinical specialties, particularly in patients with diabetes mellitus (10, 12, 13).

### Limitations

The primary limitation of this study is the small sample size, which may restrict the generalizability of the findings. Additionally, the cross-sectional design limits the ability to establish causality between microcirculatory changes and diabetes complications. Longitudinal studies with larger cohorts are needed to validate these findings and assess long-term outcomes.

## Conclusions

T2DM is associated with significant alterations in the microcirculatory bed, including endothelial dysfunction, increased sympathetic nervous system activity and elevated oxidative stress. LDF1 is an effective non-invasive method for assessing the state of microcirculation. This technique enables real-time evaluation of tissue perfusion, regulatory mechanisms, and the functional status of vascular tone. Patients with T2DM exhibit enhanced compensatory mechanisms of blood flow regulation, such as increased myogenic activity, indicating adaptive processes in response to chronic microvascular damage. LDF detects preclinical microcirculatory changes, notably decreased endothelial activity (Ae) and increased sympathetic adrenergic activity (An), reflecting impaired regulation of the vascular bed. The findings emphasize the necessity of early monitoring of microcirculatory status in patients with T2DM for timely detection of complications such as neuropathy, angiopathy, and retinopathy, as well as for therapy adjustment. LDF holds promise as a standard tool for the diagnosis and monitoring of microvascular lesions in chronic endocrine disorders, including diabetes mellitus.

## Data Availability

The datasets presented in this article are not readily available because The dataset is anonymized and available upon reasonable request. It may not be used for commercial purposes without prior permission. Any reuse should cite the original article. No personally identifiable information is included. Requests to access the datasets should be directed to zlataligonenko@gmail.com.
